# Network-level functional connectivity is associated with longitudinal tau accumulation and amyloid-dependent cognitive decline in preclinical Alzheimer’s disease

**DOI:** 10.21203/rs.3.rs-10282950/v1

**Published:** 2026-08-25

**Authors:** Mohammadali Javanray, Jonathan Gallego-Rudolf, Yara Yakoub, Ting Qiu, Frederic St-Onge, Jordana Remz, Jean-Paul Soucy, Bratislav Misic, Lune Bellec, Jacob Vogel, PREVENT-AD Research Group, Sylvia Villeneuve

**Affiliations:** McGill University; McGill University; McGill University; McGill University; McGill University; Douglas Mental Health University Institute; McGill University; Montreal Neurological Institute, McGill University; Université de Montréal; Lund University; Douglas Mental Health University Institute; McGill University

## Abstract

Amyloid-β and tau, pathological hallmarks of AD, silently accumulate years before clinical symptoms’ onset and are associated with neuronal dysfunction and cognitive impairment. Although functional connectivity has shown promise in tracking AD proteinopathy, how large-scale brain functional connections and their temporal variability relate to longitudinal pathological and cognitive changes during the preclinical stage remains unclear. Here, we examined both static and time-varying functional connectivity across brain networks in cognitively unimpaired older adults with a family history of AD, who underwent resting-state fMRI, longitudinal amyloid- and tau-PET imaging, and decade-long neuropsychological assessments. We found that lower static connectivity across multiple large-scale networks was associated with faster longitudinal tau accumulation and, selectively in individuals with elevated amyloid burden, with accelerated cognitive decline. These associations were not observed for time-varying connectivity. Together, our results demonstrate a role of static functional network organization in early pathological and cognitive progression of AD.

## Introduction

Alzheimer’s Disease (AD) is the most prevalent form of dementia affecting approximately 50 million individuals worldwide, a number projected to triple by 2050 [1]. The accumulation of amyloid-β (Aβ) and tau proteins, the pathological hallmarks of AD, begins years or even decades prior to the onset of clinical symptoms, marking the preclinical phase of the disease [2, 3]. Importantly, this preclinical phase offers a critical window for early intervention and disease prevention [4]. Given the role of Aβ and tau on brain neuronal and network-level dysfunction [5, 6] and cognitive impairment [2], slowing down the early accumulation of these pathologies is eminent towards preserving brain function in AD. Brain functional connections have shown promise in predicting the spatio-temporal pattern of AD proteinopathy [7, 8]. However, whether these network connections and their intrinsic time-dependant variabilities contribute to longitudinal AD pathology and cognitive function in the preclinical disease stage remains unclear. It is thus important to conduct studies that elaborate on these relationships at the preclinical stage of AD with the aim of better understanding brain functional networks’ role in early AD progression.

Pathology-related changes in functional connectivity estimated from resting-state functional magnetic resonance imaging (fMRI) have been reported in cognitively unimpaired individuals at risk of AD and throughout the AD continuum [9]. The default mode (DMN) and limbic networks spatially overlap with early sites of Aβ and tau accumulation [10, 11], respectively, making these networks particularly vulnerable to pathology-driven functional alterations in the early stages of the disease continuum [9, 11]. However, given that large-scale brain function arises from coordinated activity among multiple systems [12], whole-brain network functional connectivity relationship to AD proteinopathy offers a more comprehensive understanding of functional-pathological relationship in AD. Consistent with this broader perspective, in preclinical AD and mild cognitive impairment (MCI) stages, large scale systems display both hyper- and hypoconnectivity [13, 14], whereas later stages of the disease are characterized by widespread connectivity loss [15]. At the preclinical stage particularly, studies have reported hypoconnectivity [16], hyperconnectivity [17], or no association between functional connectivity and AD proteinopathy [18]. Longitudinal evidence further indicate that functional connectivity relates to the spread of AD pathology with highly connected brain regions exhibiting elevated tau levels, and functionally connected regions showing synchronized tau accumulation rates particularly at high Aβ levels [7, 8]. Given that the large-scale network communication supports cognitive processes [12], pathology-related alterations in their connectivity may represent an early functional pathway through which Aβ and tau accumulation impact cognition. Indeed, connectivity strength within the DMN and limbic networks, as well as across broader systems, has been associated with memory performance and global cognitive impairment [19, 20]. Together, these findings underscore the need for studies examining how large-scale connectivity patterns relate to both cross-sectional and longitudinal Aβ and tau accumulation, and perhaps contribute to early cognitive decline in individuals at elevated risk for AD.

Typically, rs-fMRI functional connectivity is estimated by computing the interregional coherence of blood-oxygen-level-dependent (BOLD) signal fluctuations [21]. Nevertheless, deriving such “static” measures of functional connectivity discard the intricate temporal information underlying fast dynamic changes in functional connectivity, which have been increasingly recognized as crucial for understanding the brain’s functional architecture both in healthy and pathological condition [22, 23]. Time-varying functional connectivity (TVFC) captures how connectivity patterns shift over time and has proven effective in distinguishing AD patients from healthy controls [24]. Furthermore, TVFC has shown to be sensitive to cognitive changes across the AD continuum [25], relate to AD genetic risk factors [26], and to link with both Aβ and tau pathology [18, 23]. Thus, analyzing intrinsic time-dependant functional connectivity and understanding its interplay with AD proteinopathy deposition and cognitive decline might provide further insights into early AD pathological and cognitive changes.

In this study, we used data from the PResymptomatic EValuation of Experimental or Novel Treatments for AD (PREVENT-AD) cohort, a group of cognitively unimpaired individuals at elevated risk for developing AD due to familial history of sporadic AD [27]. We tested the association between network TVFC and sFC with both cross-sectional and longitudinal Aβ and tau accumulation, as well as their association with longitudinal cognitive trajectories. Across multiple networks, stronger TVFC and sFC were associated with slower longitudinal tau accumulation, whereas associations with cognition were more subtle and tended to reflect better preserved cognitive performance over time in cognitively unimpaired individuals at risk of AD. These findings indicate that large-scale functional network organization, in its time-varying and static configuration, may contribute to reduced vulnerability to tau accumulation and preserved cognition in the preclinical stage of AD.

## Results

We studied a subset of 240 cognitively unimpaired participants from the PREVENT-AD cohort who underwent multimodal neuroimaging (Aβ and tau PET, structural MRI, and resting-state fMRI), RBANS cognitive testing, and APOE-ε4 genotyping. From this primary sample, 218 individuals passed fMRI quality control and were included into our analysis, comprising 72% females with a mean age of 68.16 years (SD = 5.19). All 218 underwent [18F]NAV4694 Aβ PET with a mean global weighted average Aβ Standard Uptake Value Ratio (SUVR) of 1.32 (SD = 0.29). Flortaucipir [18F]AV1451 tau PET was available for 216 participants with a mean SUVR of 1.16 (SD = 0.12) in a temporal meta-ROI known to accumulate tau early in the disease (see Methods for the regions). The 218 participants also completed repeated neuropsychological assessments, with a baseline mean RBANS total scale index score of 104.07 (SD = 10.07), an average of 7.33 visits per participant (SD = 2.30), and longitudinal follow-ups spanning a mean duration of 7.81 years (SD = 1.82), with a mean inter-visit interval of 1.23 years (SD = 0.75). Demographics are summarized in Table 1. For longitudinal PET measurements, 101 participants completed follow-up Aβ PET with a mean interval of 4.33 years (SD = 0.54). Of these, 95 also underwent follow-up tau PET, with a mean interval of 4.28 years (SD = 0.55). More detailed inclusion/exclusion criteria and imaging/cognitive acquisition are provided in Methods.

Summary of the main characteristics of the data utilized for our analysis from the PResymptomatic EValuation of Experimental or Novel Treatments for AD (PREVENT-AD) cohort. The table presents the key demographics of the data, as well as the mean (and SD) of the baseline global Aβ SUVR, temporal meta-ROI tau, RBANS total scale index, and time intervals between MRI and PET measurements. Abbreviations in the table are as follows: PET= Positron Emission Tomography, APOE4 = Apolipoprotein E ε4, SUVR= Standard Uptake Value Ratio, ROI= Region of Interest, fMRI= functional Magnetic Resonance Imaging, and Aβ = Amyloid Beta.

**Figure 1:**
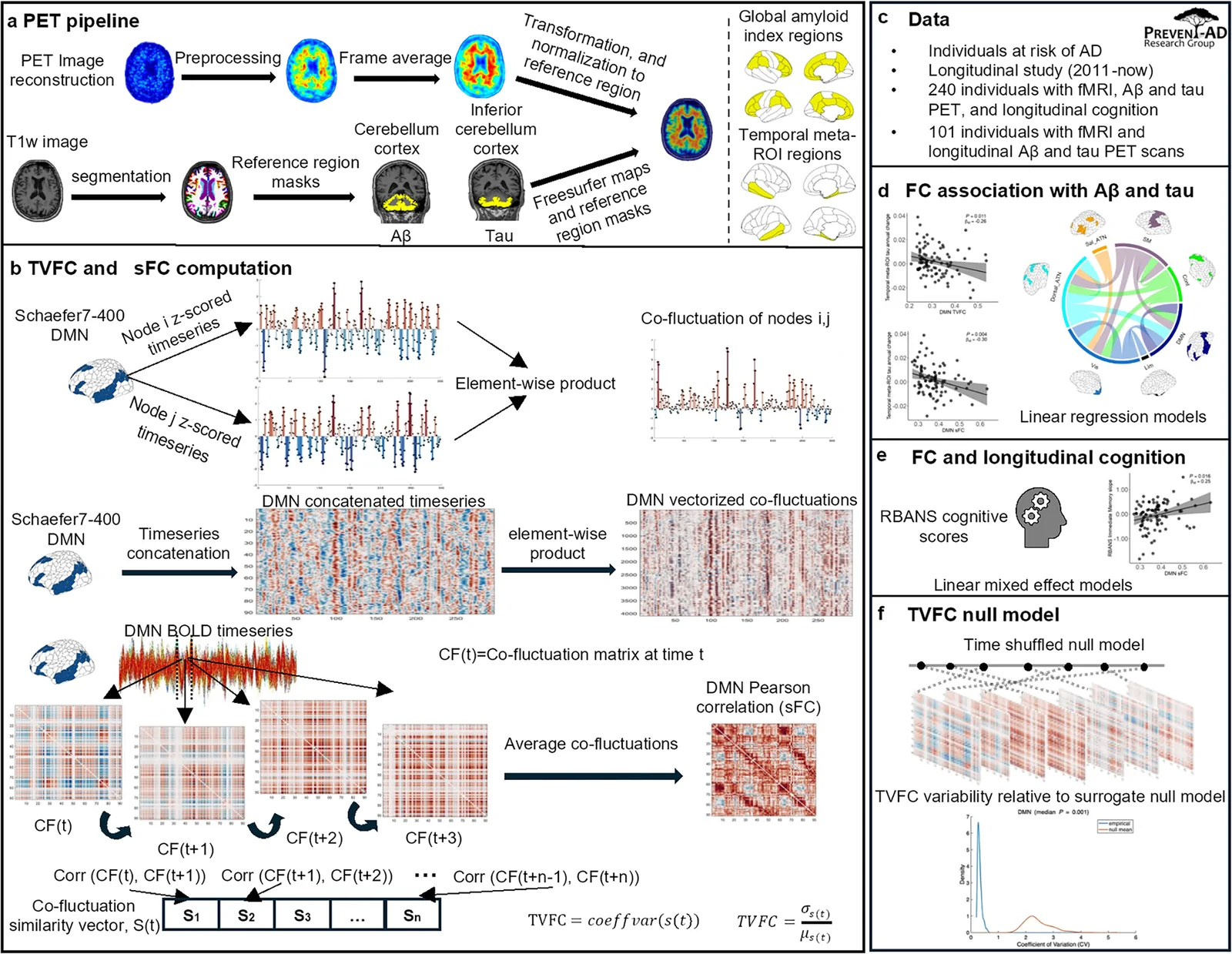
Multimodal data processing and analysis pipeline. **a**, Overview of the PET processing pipeline, including motion correction, frame averaging, spatial normalization, and extraction of global Aβ and temporal meta-ROI tau SUVR indices. **b**, Illustration of the computation of TVFC and static sFC. Edge timeseries were generated by computing the element-wise product of z-scored timeseries between all node pairs of the Schaefer7–400 atlas. For TVFC, edge timeseries were assembled into co-fluctuation matrices at each time point (CF(t)); the Pearson correlation between consecutive matrices yielded a similarity vector S(t), from which the coefficient of variation was calculated as the TVFC measure. Four example DMN co-fluctuation matrices are shown. **c**, Summary of the quantitative and descriptive characteristics of the study cohort and imaging data. **d**, Associations between functional connectivity and Aβ/tau were tested using linear regression, with results highlighted for DMN, limbic, and DMN–limbic networks given their relevance to early AD pathology. Chord plots illustrate significant associations across all Schaefer7–400 networks. **e**, Associations between functional connectivity measures and cognition (RBANS total score) were evaluated using linear mixed-effect models. **f**, Surrogate null models with time-shuffled edge timeseries co-fluctuation matrices were used to validate our estimated empirical TVFC values.

We used the edge timeseries method [28] to derive a measure of TVFC, capturing the temporal variability of brain network functional connections. sFC was computed using Pearson correlations across brain node pairs. We then examined whether TVFC and sFC were associated with cross-sectional and/or longitudinal measures of Aβ and tau, and with cognition. Linear regression models were used to compute the associations, while linear mixed effect models were used to extract the slope of cognitive changes over time. An overview of the study design and processing workflow is presented in Fig. 1.

### TVFC and sFC associations with cross-sectional Aβ and tau PET accumulations

**Figure 2:**
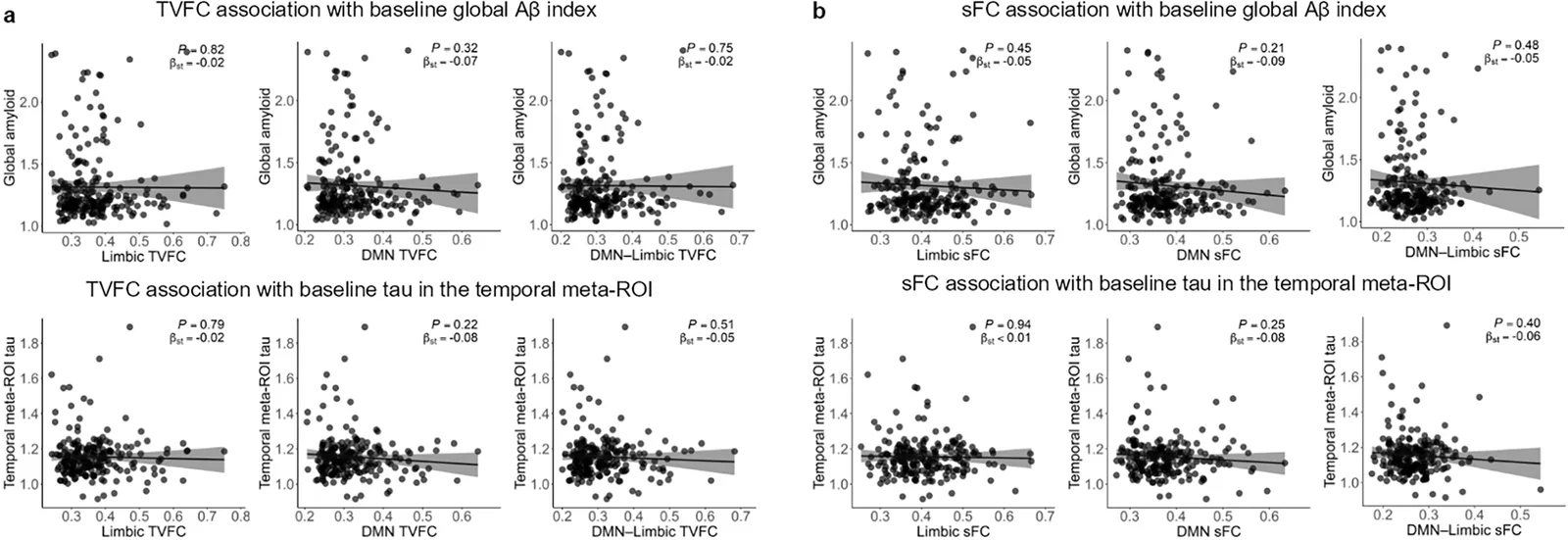
TVFC and sFC relationships with cross-sectional Aβ and tau PET. **a**, Linear regression models examining the associations between TVFC in the DMN, limbic network, and between DMN and limbic networks with cross-sectional Aβ and tau. **b,** Linear regression models with similar to panel a, testing associations of sFC with cross-sectional Aβ and tau. Shaded region around the line of best fit across all participants represents the 95% confidence intervals.

Given the early vulnerability of the DMN and limbic networks to AD pathology, our analyses first targeted these systems as a priori networks of interest. We tested whether TVFC and sFC within and between these early-vulnerable networks relate to longitudinal and cross-sectional Aβ and tau burden (Fig. 2a–b). We assessed the relationship between TVFC and sFC and continuous measurements of AD pathology using linear regression analyses while including age, sex, mean framewise displacement (FD), and the time interval between fMRI and PET scans as covariates. Cross-sectionally, we found no significant associations between Aβ or tau burden and either within- or between-network DMN and limbic TVFC or sFC (Fig. 2a–b).

Across individuals, elevated Aβ burden was rarely accompanied by high TVFC values in DMN, limbic and between DMN and limbic networks. Collectively, higher TVFC values were largely restricted to individuals with lower Aβ levels with a relative absence of high TVFC values at higher Aβ burden (Fig. 2a). Similar distributional pattern was observed for tau burden (Fig. 2a). The patterns observed for sFC were largely similar to those identified for TVFC in relation to Aβ and tau burden, though not as pronounced (Fig. 2b). To examine whether Aβ burden influenced the association between TVFC and longitudinal tau accumulation, Aβ was included as an interaction term in regression models relating TVFC to cross-sectional tau accumulation; however, no significant TVFC × Aβ interaction effects were observed on the tested associations.

We then expanded our analyses to include all the seven Schaefer7–400 networks and their between-network connections, providing a comprehensive, exploratory assessment of the broader topographical organization of functional connectivity-proteinopathy relationships (Supplementary Fig. 1a-d). Linear regression analysis similar to the ones used for DMN and limbic were used to assess the relationship between sFC, TVFC across all the networks. Similar to the DMN and limbic, no association was observed between cross-sectional AD proteinopathy with either TVFC or sFC (Supplementary Fig. 1a-d) across whole brain network connections. All analyses were replicated using resting-state networks derived from the Schaefer7–200 parcellation, yielding consistent null associations (Supplementary Fig. 2a-d). When conducting similar analysis while restricting the fMRI-PET time intervals to less than two years (Supplementary Fig. 3a-d), and one year respectively (Supplementary Fig. 4a-d), no associations were either found between the two static and time-varying connectivity measures and cross-sectional pathology levels.

### TVFC and sFC associations with longitudinal Aβ and tau PET accumulations

**Figure 3:**
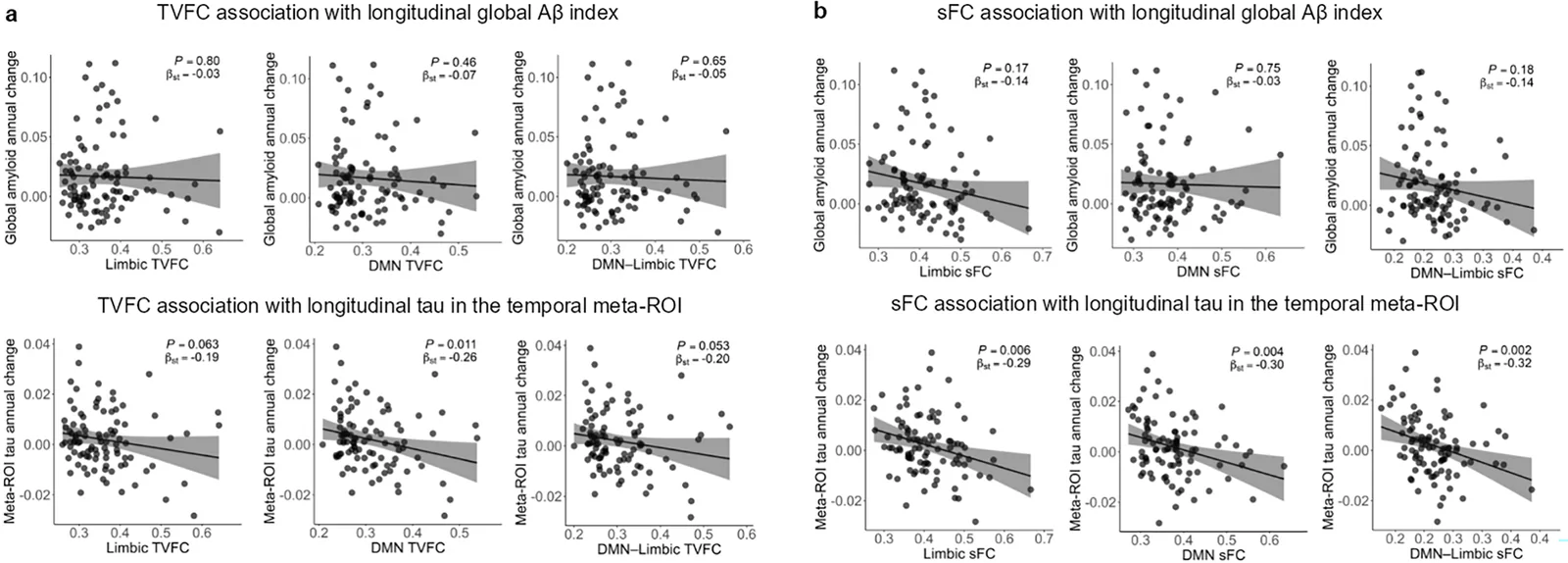
TVFC and sFC relationships with longitudinal Aβ and tau PET. **a**, Associations of the annual changes in global Aβ and tau SUVR with TVFC examined using linear regression models. **b**, Equivalent linear regression models to panel a performed to assess the associations between sFC and the annual change in global Aβ and temporal meta-ROI tau SUVR. Shaded region around the line of best fit across all participants represents the 95% confidence intervals.

We used the subset of participants who underwent both baseline and follow-up Aβ and tau PET scans to examine whether sFC and TVFC were associated with longitudinal changes in AD pathology (see Methods). The annual rates of change in Aβ and tau pathology were calculated as the difference in SUVR values between baseline and follow-up scans, normalized by the inter-scan time interval. Linear regression models, adjusted for age, sex, PET-fMRI interval, and mean FD, demonstrated that lower TVFC within the DMN was associated with faster longitudinal tau accumulation in the temporal meta-ROI regions (Fig. 3a). In contrast, no significant associations were found for TVFC within the limbic or between the DMN and limbic networks with longitudinal tau accumulations (Fig. 3a). However, lower sFC within the DMN, limbic, and between the DMN and limbic networks was consistently associated with faster annual rate of tau accumulation in the temporal meta-ROI regions (Fig. 3b). No further associations were found between either TVFC or sFC and the annual rate of change in global Aβ burden in the DMN, limbic and between DMN and limbic networks (Fig. 3a–b). All these longitudinal findings were consistently replicated when using Schaefer7–200 parcellation (Supplementary Fig. 5a-d). To examine whether Aβ burden influenced the association between TVFC and longitudinal tau accumulation, Aβ was included as an interaction term in regression models relating TVFC to longitudinal tau accumulation; however, no significant TVFC × Aβ interaction effects were observed.

### Whole brain Schaefer7–400 network TVFC and sFC associations with longitudinal Aβ and tau PET accumulations

**Figure 4:**
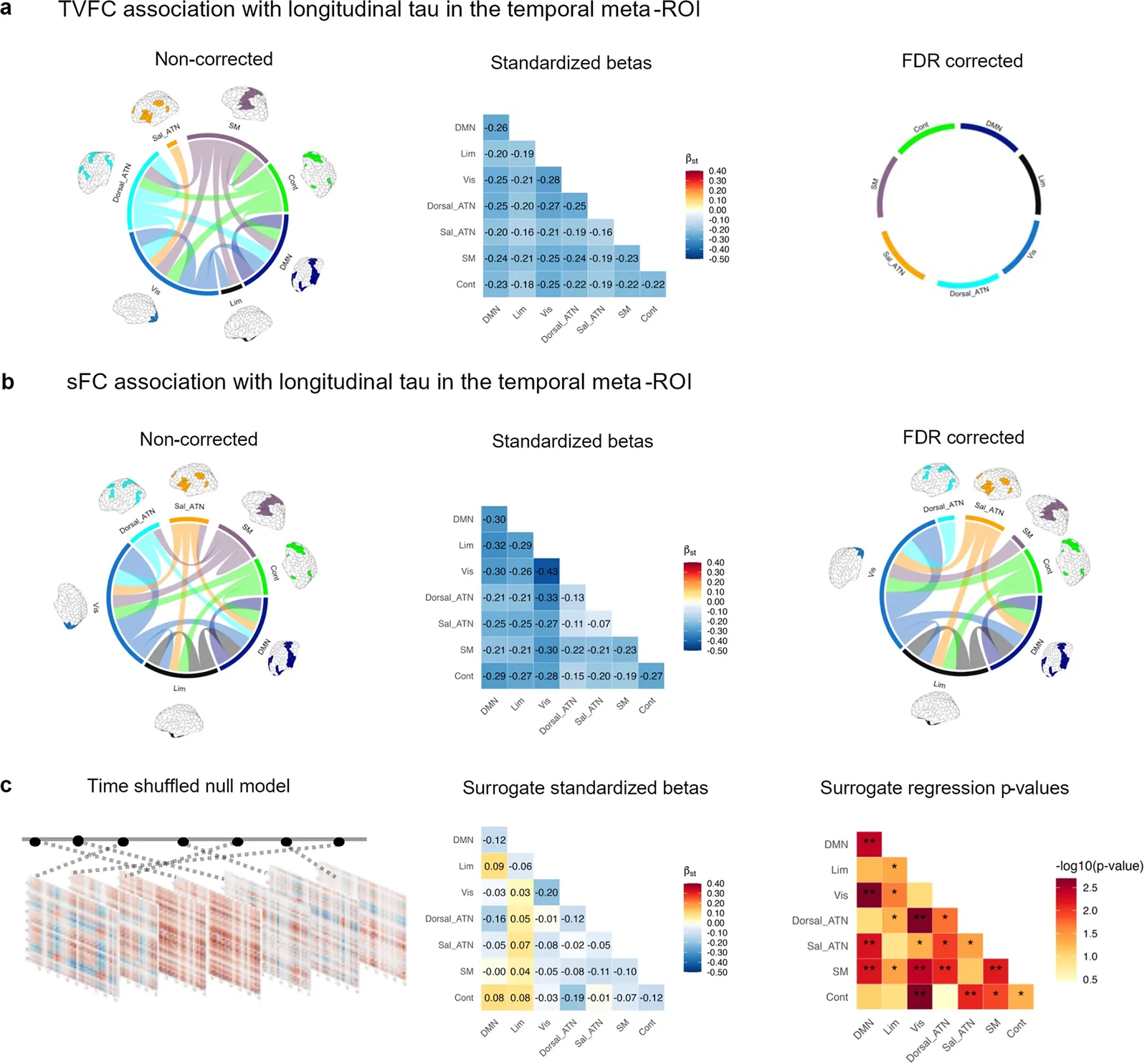
TVFC and sFC relationship with longitudinal tau PET, whole brain networks analysis. Associations between whole-brain functional connectivity measures and longitudinal tau accumulation in temporal meta-ROI regions across Schaefer 7–400 networks. **a,** Chord plots and heatmap depicting associations between TVFC and longitudinal tau accumulation. The first chord plot shows significant associations (p < 0.05), with line thickness reflecting statistical significance (lower p-values = thicker lines) and colors denoting networks: dark blue, default mode; black, limbic; sky blue, dorsal attention; purple, somatomotor; green, control; blue, visual. The heatmap displays standardized beta coefficients from linear regression models (controlling for age, sex, mean FD, and fMRI-PET interval). The second chord plot shows FDR-corrected associations, none of which survived correction. **b,** Analogous analysis for sFC across the same network parcellation, chord plots and heatmap follow identical conventions as in panel a, with the second chord plot showing associations surviving FDR correction across 28 within- and between-network tests. **c,** The left schematic is a representation of the time shuffled null model used to validate TVFC temporal structure. The central heatmap shows mean standardized beta coefficients averaged across surrogate realizations, reflecting expected association strength under the null model. The right heatmap displays Monte Carlo p-values comparing empirical to surrogate-derived null distributions; asterisks denote significance (*p < 0.05, **p < 0.01). All regression models controlled for age, sex, mean FD, and fMRI-PET scan interval. Abbreviations: DMN, Default Mode Network; Lim, Limbic; Vis, Visual; Sal_ATN, Salience/Ventral Attention; Dorsal_ATN, Dorsal Attention; Cont, Control; SM, Somatomotor.

We further took into account the interconnectedness of brain networks and expanded our analyses to all the seven Schaefer7–400 networks and their between-network connections to investigate the functional connectivity and pathology relationships in a more comprehensive and data driven approach. We found significant associations across several resting-state networks including visual, dorsal attention, control, and somatomotor systems where lower TVFC was associated with faster longitudinal rate of tau accumulation (Fig. 4a). However, when correcting for multiple comparison considering the 28 between and within network analysis performed, no association between TVFC and longitudinal tau accumulation survived the corrected significance threshold (p < 0.05) (Fig. 4a). Consistent with the DMN and limbic networks, when investigating the associations between the annual Aβ rate of change and TVFC in all the seven Schaefer7–400 networks and their between-network connections, no association was observed (Supplementary Fig. 6a). Results were highly consistent when using Schaefer7–200 parcellation (Supplementary Fig. 5a, c).

Examining sFC across all within- and between-network resting-state functional connections and longitudinal tau accumulation, we found that lower sFC was associated with faster annual tau accumulation (Fig. 4b). Specifically, within visual network connectivity again demonstrated the strongest association with longitudinal tau accumulation, corresponding to the lowest Akaike Information Criterion (AIC) value and the highest standardized beta coefficient. FDR correction was further applied when expanding our analysis to the full network analysis. Several within- and between-network sFC associations with longitudinal tau accumulation survived multiple comparison including the DMN, limbic, salience and dorsal attention, visual, limbic, control, and somatomotor network connections (Fig. 4b). Similar to TVFC, no significant association was found between sFC and longitudinal Aβ (Supplementary Fig. 6b). Consistent findings observed when using Schaefer7–200 parcellation (Supplementary Fig. 5b, d).

To confirm that the temporal variability captured by TVFC reflected genuine temporal structure rather than random fluctuations, we compared empirical TVFC estimates against time-shuffled surrogate models (Fig. 4c). The applied surrogate model disrupts the intrinsic temporal structure of functional connectivity while preserving its overall distributional properties. By comparing TVFC derived from empirical data to that obtained from randomly shuffled functional connectivity co-fluctuation timeseries, we assessed whether the observed temporal variability exceeds that expected by chance. A significant difference between empirical and surrogate-derived TVFC indicates that the measured dynamics reflect genuine, structured temporal variability in functional connectivity, rather than random fluctuations. Across networks, median subject-level p-values were significant (median of 0.001 and mean of 0.003), indicating consistent deviations of empirical TVFC from the corresponding surrogate-derived null means, and robust differences between the empirical and null model derivatives (Supplementary Fig. 7). Next, we evaluated whether empirically significant TVFC and longitudinal tau associations persisted under temporally shuffled null models. Of the 28 within- and between-network pairs tested, 18 exhibited significant empirical associations with longitudinal tau accumulation (p < 0.05). Among these, 14 associations (78%) remained significant when compared against surrogate-derived regression coefficients using the Monte Carlo test (p < 0.05). TVFC between DMN and dorsal attention, DMN and control, within visual, and between dorsal attention and control networks, did not show significant differences between empirical and surrogate-derived regression coefficients despite being significant in the empirical model (Fig. 4c).

### TVFC and sFC associations with cross-sectional cognition

**Figure 5:**
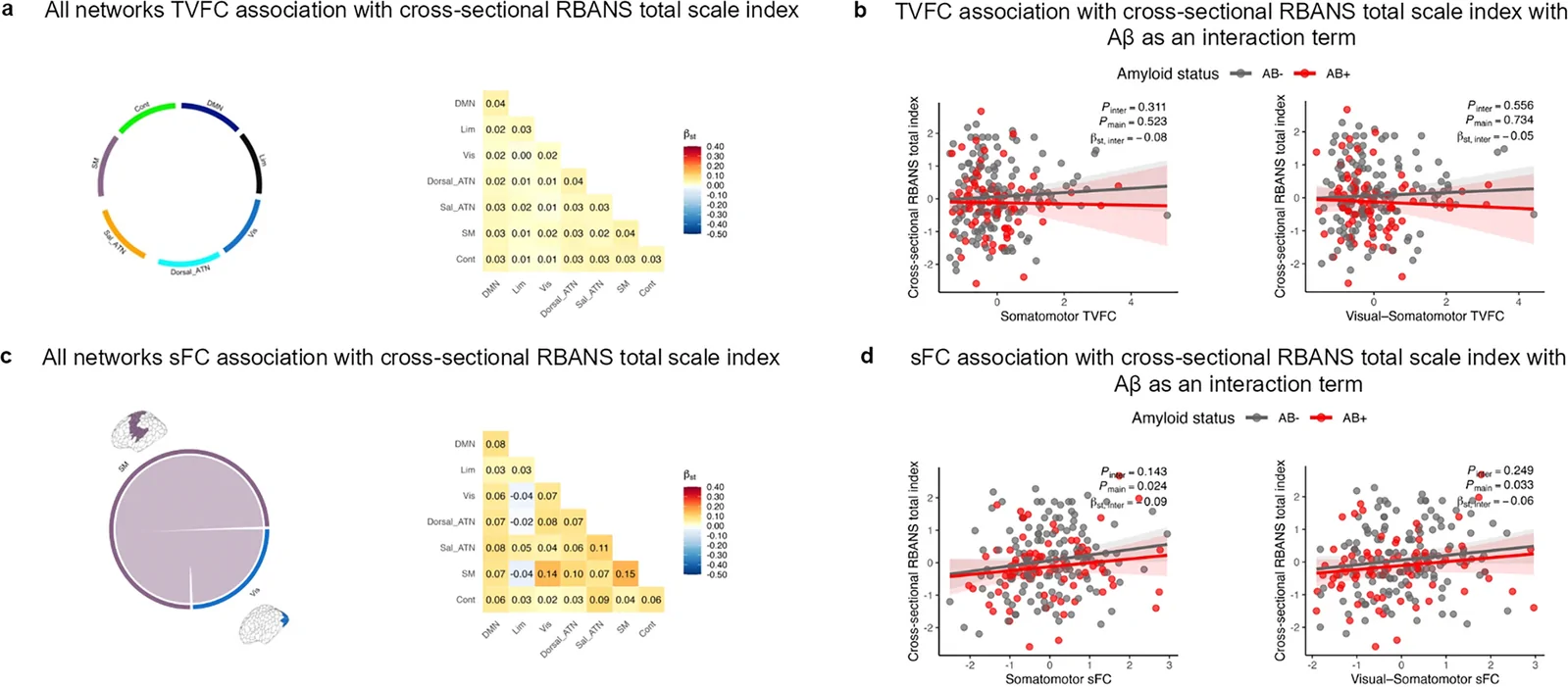
TVFC and sFC relationship with cross-sectional RBANS total cognitive score. Associations between functional connectivity measures and cross-sectional RBANS total score across Schaefer 7–400 networks, with and without Aβ moderation. All regression models controlled for sex, education, and mean FD. **a,** Chord plot and heatmap depicting associations between TVFC and RBANS total score. The empty chord plot indicates no significant association (p > 0.05) for TVFC; the heatmap displays corresponding standardized beta coefficients. **b**, Scatter plots illustrating the null interaction effect of Aβ on the TVFC and RBANS association for connections showing significant main effects in either TVFC or sFC analyses. P_inter_ and P_main_ denote interaction and main effect p-values, respectively; β_st,inter_ denotes the standardized interaction beta. Aβ dichotomization was applied for visualization only; statistical estimates are derived from continuous Aβ values. **c**, A parallel analysis for sFC, following identical chord plot and heatmap structure as in a. The chord plot shows significant associations (p < 0.05), with line thickness reflecting statistical significance and colors denoting networks: purple, somatomotor; blue, visual. **d**, Displaying the null interaction effect of Aβ on the sFC and RBANS association. Shaded region around the line of best fit across all participants represents the 95% confidence intervals. Abbreviations: DMN, Default Mode Network; Lim, Limbic; Vis, Visual; Sal_ATN, Salience/Ventral Attention; Dorsal_ATN, Dorsal Attention; Cont, Control; SM, Somatomotor.

Given the involvement of brain functional networks in diverse cognitive processes, we examined how TVFC and sFC are associated with cognitive performances in early AD. We initially tested the RBANS total composite score’s association with TVFC and sFC using linear regression models, adjusting for sex, education, and mean FD. When exploring the DMN and limbic networks, neither sFC nor TVFC were associated with RBANS total composite score. Extending the analyses to whole brain within- and between-network connections revealed no significant associations between TVFC in other functional networks and cognition (Fig. 5a). However, stronger sFC within somatomotor (standardized β = 0.15, p = 0.024), and between the visual and somatomotor networks (standardized β = 0.14, p = 0.033), were associated with higher total RBANS cognitive score (Fig. 5c). When correcting for multiple comparisons using FDR correction and considering the 28 within and between network analysis, these associations did not survive. When including baseline Aβ in our models as an interaction term to assess its effect on the association between either TVFC or sFC and cross-sectional cognition, we found no significant moderation effect (Fig. 5b, d).

### TVFC and sFC associations with a decade-long longitudinal cognitive trajectory

To better understand large-scale network connectivity relationship to longitudinal cognitive trajectories in preclinical AD, we examined TVFC and sFC associations with longitudinal changes in RBANS cognitive scores. Individual cognitive change rates were derived from linear mixed effect models, and fed to linear regression models adjusted for sex, education, and mean FD, to test whether sFC or TVFC measures were associated with the cognitive slopes. We first focused on the DMN, limbic network, and their between network connections, given their early vulnerability to AD pathology, and then we extended our analysis to whole brain network connections.

**Figure 6:**
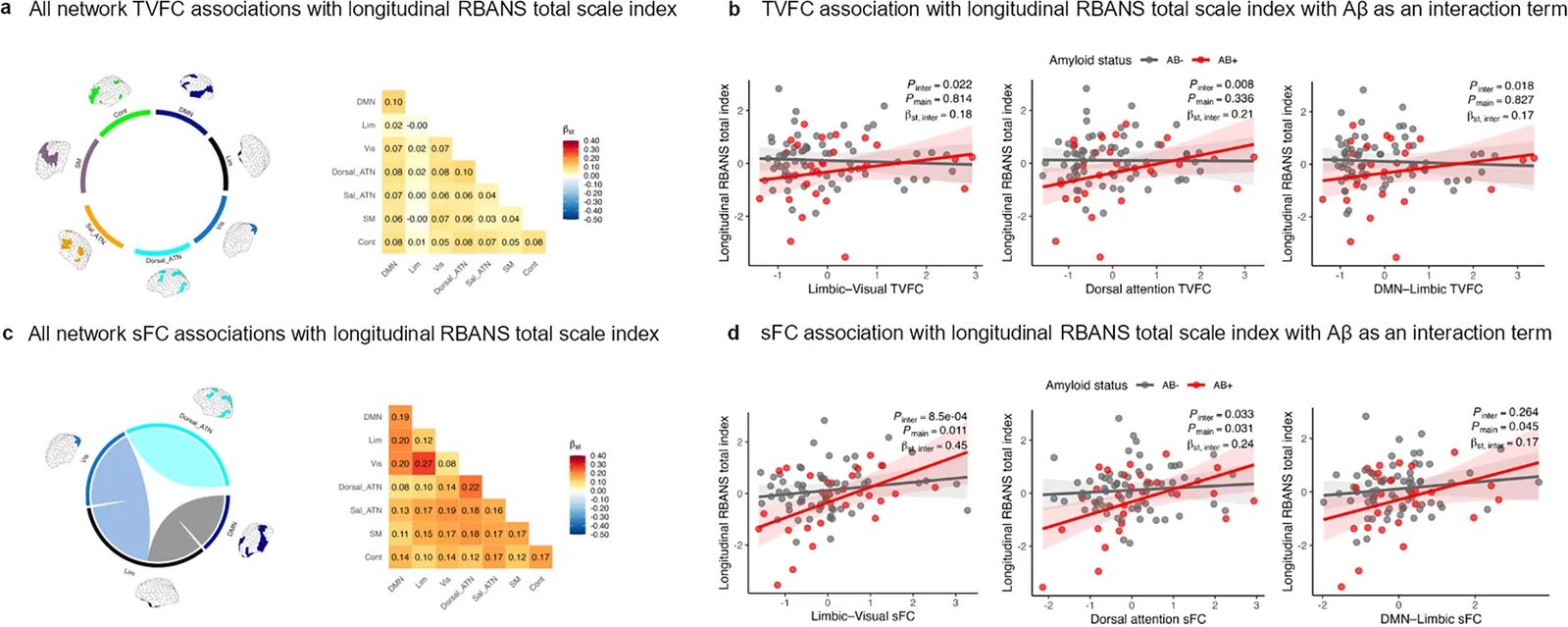
TVFC and sFC relationship with the decade-long longitudinal cognition. Associations between functional connectivity measures and longitudinal RBANS total score across Schaefer 7–400 networks, with and without Aβ moderation. Only connections with significant main effects are shown for the Aβ interaction analyses. **a,** Chord plot and heatmap depicting associations between TVFC and longitudinal RBANS total score. The empty chord plot indicates no significant association (p > 0.05); the heatmap displays corresponding standardized beta coefficients. **b**, Scatter plots illustrating the Aβ interaction effect on the TVFC and longitudinal RBANS association for the three connections showing significant main effects in either TVFC or sFC analyses. P_inter_ and P_main_ denote interaction and main effect p-values, respectively; β_st,inter_ denotes the standardized interaction beta. Aβ dichotomization was applied for visualization only; statistical estimates are derived from continuous Aβ values. **c**, A parallel analysis for sFC, following identical chord plot and heatmap structure as in panel a. The chord plot shows significant associations (p < 0.05), with line thickness reflecting statistical significance and colors denoting networks: dark blue, DMN; black, limbic; sky blue, dorsal attention; blue, visual. **d**, Displaying the Aβ interaction effect on the sFC and longitudinal RBANS association. Shaded region around the line of best fit across all participants represents the 95% confidence intervals. Abbreviations in the figure are as follows: DMN = default mode network, Lim = limbic network, Vis = visual network, Sal_ATN = salience/ventral attention network, Dorsal_ATN = dorsal attention network, Cont = control network, and SM = somatomotor network.

Lower sFC between the default mode and limbic networks was associated with faster decline in the RBANS total cognitive score (standardized β = 0.20, p = 0.045), whereas no significant associations were observed between TVFC and longitudinal RBANS total cognitive performance. Expanding the analysis to all the Schaefer7–400 networks, lower sFC between visual and limbic networks (standardized β = 0.27, p = 0.011) and within dorsal attention network (standardized β = 0.22, p = 0.031) were also associated with faster decline in longitudinal RBANS total cognitive score, while TVFC showed no association with longitudinal cognition (Fig. 6a, c). However, the observed significant associations did not survive correction for multiple comparisons using FDR correction.

We further examined whether Aβ burden modified the association of functional connectivity and longitudinal RBANS total index cognitive trajectories by adding baseline Aβ as an interaction term to our previous models. Although no main effect was observed between TVFC and longitudinal RBANS cognitive total score trajectories, we identified an interaction with baseline Aβ across all within- and between-network TVFC measures. Our findings revealed lower TVFC to be associated with faster cognitive decline in individuals with higher Aβ burden (Fig. 6b, Supplementary Fig. 8). Baseline Aβ similarly moderated the association between sFC and longitudinal trajectories of RBANS total scale scores. These associations were mostly restricted to limbic intra- and inter-network connections, with the strongest interaction involving sFC between the limbic and visual networks (standardized β = 0.45, *P*_*inter*_ < 0.001, *P*_*inter, FDR*_ = 0.024; Fig. 6d). Additional FDR-significant interactions for sFC and longitudinal RBANS total scale decline were observed within the limbic network (standardized β = 0.33, P_inter = 0.003, P_inter,FDR = 0.026, Supplementary Fig. 9) and between the limbic and salience networks (standardized β = 0.40, P_inter = 0.003, P_inter,FDR = 0.026, Supplementary Fig. 9). These findings further emphasize the central role of limbic connectivity in moderating the relationship between Aβ burden and cognitive decline. The direction of these effects mirrored those observed for TVFC. To further determine whether the observed TVFC×Aβ interaction effect on longitudinal cognition depended on the inherent temporal structure, we tested these associations against temporally shuffled null model (see Methods). Monte Carlo comparisons revealed that the empirical interaction coefficients did not significantly differ from those obtained using surrogate-derived TVFC estimates. These findings suggest that the moderation effects on cognition were not specifically driven by the temporal organization of TVFC.

Repeating these analyses using TVFC and sFC measures derived from the Schaefer7–200 atlas yielded highly comparable results, with lower sFC showing associations with faster decline in longitudinal RBANS total cognitive score, supporting the robustness of these findings using a second functional parcellation resolution (Supplementary Fig. 10). Similar to our findings with Schaefer7–400 parcellation, we noticed lower sFC being associated with faster cognitive decline in individuals with higher Aβ burden (Supplementary Fig. 11,12). Together, our results suggest that lower large-scale network sFC relate to faster longitudinal cognitive decline in individuals with higher level of baseline Aβ.

## Discussion

Our aim was to investigate the relationship of brain functional connectivity with longitudinal AD proteinopathy, and cognitive trajectories in the preclinical stage of AD, to clarify the potential contribution of large-scale network function to early pathological and cognitive changes. Leveraging longitudinal Aβ and tau PET imaging, decade-long neuropsychological assessments, and resting-state fMRI-derived connectomes from the PREVENT-AD cohort, we investigated both TVFC and sFC relationship to AD longitudinal proteinopathy accumulation and cognitive decline. Across multiple resting-state networks, we found that lower sFC was associated with faster longitudinal tau accumulation, and with accelerated cognitive decline in individuals with elevated Aβ burden. In contrast, TVFC was not associated with longitudinal tau accumulation or cognitive decline. These findings suggest that reduced intrinsic connectivity within and between large-scale functional systems may contribute to vulnerability to tau propagation and cognitive decline in the preclinical stage of AD.

Cross-sectionally, we observed no associations between TVFC or sFC with either Aβ or tau pathology. This likely reflects the cognitively unimpaired status of the individuals studied, in whom AD pathology is typically still limited [2] and functional consequences remain subtle despite elevated risk. These findings are in line with previous research, some of which also reported no association between functional connectivity and Aβ in cognitively unimpaired individuals [18, 29]. Cross-sectional associations between both sFC and TVFC and cognitive performance were mild and did not survive FDR correction. These could be explained by the fact that only cognitively unimpaired individuals were included in cross-sectional analyses and that inter-individual differences not-related to AD, might overcome early disease-specific impairments.

Longitudinally, lower sFC across multiple large-scale brain systems was associated with faster tau accumulation over time. Although associations between lower TVFC and faster tau accumulation were observed across distributed networks and exceeded those computed from time-shuffled null models, these effects did not survive correction for multiple comparisons. The observed associations were evident within and between the default mode and limbic networks, networks known to exhibit both functional disruption and tau deposition early in AD [9]. They were also found in all other tested networks, suggesting a whole brain “dysconnectivity” rather than network-specific findings. Reduced or less functionally connected patterns have been found in individuals with AD dementia [15], but results in patients in the mild cognitive impairment and preclinical phases of AD have been inconsistent [13, 14].

Under normal conditions, tau is a protein that binds to microtubules and plays a key role in microtubule stability, axonal transport, and neurite outgrowth [30]. In tauopathies, tau loses its ability to bind microtubules and detached tau proteins can misfold, a phenomenon that is probably accelerated by Aβ [31]. In-vivo group-level fMRI studies have found that brain regions that are more strongly connected tend to accumulate tau-PET more synchronously over time [7, 8]. Seed based analytic approaches have also shown that using tau-PET epicenters as a seeding point for resting state fMRI can inform the spatiotemporal localisation of tau [7, 8], supporting the hypothesis that tauopathies spread along neuroanatomically connected regions in a “prion-like” manner [32]. Interestingly, in regions with low tau-PET uptake, an inverse association has however been found, such that lower connectivity was associated with increased tau-PET binding in strongly connected regions [8]. Increasing tau burden has also been associated with reduced functional connectivity between key hubs of the default mode and limbic networks [33]. Our results add to this literature by showing that, at the whole brain connectome level, lower connectivity within and between large scale networks is associated with increased tau accumulation in individuals who are still cognitively unimpaired. It is unknown whether, at the very early disease stage, lower functional connectivity reflects a vulnerability toward future tau accumulation, or if global brain “dysconnection” is a consequence of AD pathological changes, or both. As a take home, while the strength of the functional connectivity between two regions can predict where tau will spread, reduced functional connectivity at a global level is associated with faster tau accumulation in asymptomatic individuals.

In contrast to our robust associations between sFC and tau progression, we did not observe significant relationships between either TVFC or sFC and global Aβ burden or rate of change. These findings are in line with other studies demonstrating similar null associations between functional connectivity and Aβ [18, 29]. Electrophysiological findings also reported weak coupling between Aβ load and resting-state electroencephalography functional connectivity [34], and baseline magnetoencephalography functional connectivity failing to predict longitudinal Aβ accumulation [35]. Previous findings from our group including an overlapping 104 participants and using magnetoencephalography however suggested an Aβ-related increase in brain activity preceding a tau-related decrease in brain activity [6]. Together, our findings along with other studies, suggest that Aβ is more closely related to brain activity than large-scale functional network organization during preclinical stages of the disease.

We further observed that lower sFC within limbic network and between limbic and visual and salience attention networks, was associated with faster cognitive decline, an effect that was driven by individuals with elevated Aβ. These findings align with prior work showing that reductions in functional connectivity, particularly between memory-relevant hubs such as the posterior cingulate cortex, medial prefrontal cortex, and hippocampus were related to lower cognitive performance in cognitively normal older adults and early AD [33]. Such associations were not found at the whole group level when using TVFC as they did not significantly exceed those observed under time-shuffled surrogate models, suggesting that TVFC’s moderation effect on cognition may not be specifically driven by the preserved temporal organization of TVFC. Our findings with sFC echo evidence coming from AD [36], and also other neurodegenerative [37] and neurodevelopmental [38] diseases, suggesting that reduced functional connectivity is associated with worse cognitive performances. Finally, in preclinical AD, higher functional connectivity has often been suggested as a compensatory mechanism that allows maintaining normal cognition despite the presence of pathology [39].

Although resting-state fMRI functional connectivity is inherently time-varying [22], TVFC was not associated with longitudinal tau accumulation or cognitive decline after correction for multiple comparisons or in comparison to surrogate models. In contrast, lower sFC across distributed networks was associated with faster tau accumulation and cognitive decline. This divergence likely reflects fundamental differences in the biological and temporal scales captured by these measures [40]. TVFC indexes moment-to-moment reconfigurations in functional coupling, whereas sFC represents a time-averaged organization of large-scale networks that may be more closely linked to slow, cumulative processes such as tau propagation and long-term cognitive change. These findings suggest that, in the preclinical stage of AD, static network organization may be more relevant to disease-related processes than short-term temporal fluctuations.

Several limitations should be considered when interpreting these findings. Resting-state fMRI is inherently susceptible to physiological and motion-related noise, which can influence functional connectivity estimates [41]. To mitigate these effects, we implemented a rigorous denoising strategy and additionally included mean FD as a covariate in our models to account for residual motion effects. For TVFC specifically, we validated our empirical estimates against a time-shuffled null model that disrupts temporal dependencies while preserving distributional properties, and observed marked differences between empirical and null data, supporting the presence of meaningful temporal structure. Nevertheless, it is important to note that temporal variability of co-fluctuations represents only one of several approaches used to characterize TVFC, and alternative methodologies may capture complementary aspects of dynamic functional organization [40]. To further assess robustness, we replicated our analyses using a different parcellation resolution and observed consistent patterns of association, supporting reproducibility across atlas scales. From a pathological perspective, the present cohort is characterized by low baseline tau burden and modest longitudinal accumulation. Finally, while we sought to minimize the impact of known off-target flortaucipir binding by excluding regions such as the hippocampus and basal ganglia [42], residual off-target signal may still be present elsewhere in the brain and cannot be fully eliminated. Additionally, we used Schaefer atlas parcellation in our analysis which was derived from functional connectivity patterns in large samples of young, healthy adults [43]. Given that aging is associated with progressive alterations in network organization [44], the canonical network boundaries defined in young adults may not fully capture the intrinsic functional architecture of older individuals at risk. Future work using subject-specific or age-adapted parcellations may take this concern into account.

Collectively, our findings indicate that lower sFC across several large-scale networks is associated with faster tau accumulation and cognitive decline in the preclinical stage of AD. However, TVFC did not show robust associations with longitudinal tau or cognition, suggesting a limited contribution of temporal dynamics in this context. Lower large-scale brain sFC might indicate vulnerability to tau accumulation, or more plausibly that by the time we can capture tau pathology with PET, serval AD-related brain processes are already in play, and that whole brain dysconnectivity is one of these processes.

## Methods

### Participants

Participants were recruited from the PREVENT-AD cohort [45], a cohort of cognitively unimpaired individuals with elevated risk of developing AD due to a family history of AD involving at least one parent or multiple siblings. PREVENT-AD is an ongoing longitudinal study that started in 2011 and comprises 387 participants. Participants were tested for being cognitively unimpaired at the time of enrollment and must have had a parent or two siblings who were diagnosed with AD-like dementia to be enrolled. The inclusion criteria were as follows: (1) being cognitively unimpaired; (2) having a parent or two siblings diagnosed with AD-like dementia, and therefore being at increased risk of sporadic AD; (3) age 60 years old and above or between 55 and 59 if within 15 years of their affected family member’s age at symptom onset; (3) being free of major neurological and psychiatric diseases; and (4) to have a minimum of 6 years formal education. The Clinical Dementia Rating (CDR) and the Montreal Cognitive Assessment (MoCA) were performed at enrolment to assess normal cognition. If these initial assessments yielded uncertain results specifically, a CDR score of 0.5 or a MoCA score of 26 or below, a certified neuropsychologist conducted a more detailed neuropsychological evaluation to confirm normal cognition. Additionally, all participants completed the Repeatable Battery for the Assessment of Neuropsychological Status (RBANS) [46] at baseline. Participants whose RBANS scores were lower than expected given their age and/or educational background also completed a clinical neuropsychological battery to confirm normal cognition. A total of 244 individuals underwent Aβ-PET imaging and 242 underwent tau-PET imaging (all participants who did a tau scan were scanned for Aβ). From these individuals, 4 were not included in the study because they were classified as MCI at the time of their PET scan, 3 did not have a fMRI scan and 19 had excessive motion during their fMRI scans. The final sample was therefore 218 individuals with Aβ, and 216 individuals with tau. Among these participants, 101 completed a follow-up Aβ-PET scan with an average interval of 4.34 years (SD = 0.54). Of these, 95 also underwent follow-up tau-PET imaging with an average interval of 4.28 years (SD = 0.55).

### Positron emission tomography (PET) acquisition

PET scans of the PREVENT-AD cohort were acquired at the McConnell Brain Imaging Center (BIC) located in Montreal Neurological Institute using a PET scanner with a High-Resolution Research Tomograph (HRRT) human brain camera developed and commercialized by CTI/Siemens. For the detection of Aβ, [18F]NAV4694 tracer was injected (injected dose ≈ 6 mCi), and scans were further acquired 40–70 mins after the injection. To assess the level of tau in the brain, Flortaucipir [18F]AV1451 tracer was injected (injected dose ≈ 10 mCi) and scans were acquired 80–100 mins post-injection. The first set of PET scans were performed between 2017–2019 and the second round of them were acquired between 2021–2023 for the sum of 244 baseline scans and 104 longitudinal follow ups.

### PET preprocessing

The PET images were preprocessed using an in-house standard PET pipeline which is publicly available (https://github.com/villeneuvelab/vlpp). To summarize the preprocessing steps, images were first converted from minc to nifti. The obtained nifti images were smoothed using a 6-mm^3^ Gaussian kernel and motion correction was applied using a two-pass approach in SPM. In the first pass, all the image volumes were aligned to the first volume in the series, and an average of the realigned images was created. Then, all the images were aligned to this average template. The average template was then co-registered to the same participant’s T1w image which was previously parcellated with the Desikan-Killiany atlas using the FreeSurfer version 5.3 [47]. Non-brain tissue was removed to reduce contamination by non-grey and non-WM voxels. To compare the tracer uptake between individuals, we computed the standardized uptake value ratio (SUVR) by dividing the standardized uptake value from all voxels by the cerebellar gray matter and the inferior cerebellum gray matter for Aβ [48] and tau [49] scans, respectively. Images were further inspected for unusual, elevated binding in their reference region, and for registration errors. To derive an index of global Aβ, we averaged the bilateral Aβ SUVR values within the regions that are known to accumulate Aβ at the early stages of AD (medial orbitofrontal, rostral anterior cingulate, posterior cingulate, precuneus, rostral middle frontal, superior frontal, and inferior parietal cortices) [50, 51]. For quantifying tau pathology, we averaged the bilateral tau SUVR across the following early tau-accumulating regions [52]: entorhinal cortex, amygdala, parahippocampal gyrus, fusiform gyrus, and inferior and middle temporal cortex regions of interest (ROIs) as the temporal meta-ROI tau SUVR [53]. A brief representation of the process is illustrated in Fig. 1 (Fig. 1a). The slope of Aβ and tau changes over time were computed by calculating the differences in the SUVR values of the baseline and follow up scans divided by their time interval in years. Participants were further classified based on their level of pathology. For Aβ classification, participants were grouped as Aβ-negative (SUVR < 1.26) or Aβ-positive (SUVR > 1.26) based on their global Aβ level using a threshold set at the average of two estimates: one from a Gaussian mixture model fitting two distributions (SUVR = 1.33) [54], and the other one derived from 2 SD above the mean of 11 young individuals (< 40 years old) who underwent the same PET protocol (SUVR = 1.12) [55].

### MRI acquisition

MRI scans were acquired using a 3T Siemens Trio scanner at the Cerebral Imaging Center (CIC) of the Douglas Mental Health University Institute (Montreal, Canada). Details of the scanning protocol have been reported previously [56]. Briefly, we used a Magnetization Prepared Rapid Gradient Echo Imaging (MPRAGE) sequence to acquire the T1-weighted (T1w) structural data. The Structural T1-weighted images were obtained with the following specified parameters: TR = 2300ms, TE = 2.98 ms, number of slices = 176, slice thickness = 1 mm, inversion time (TI) = 900 ms, flip angle = 9°, Field of View (FOV) = 256 × 240 × 176 mm, phase encode A-P, GRAPPA acceleration factor = 2. Two consecutives runs (150 volumes; 5:04 minutes length) of resting-state fMRI scans were acquired using a blood-oxygen-level-dependent (BOLD) sensitive, single-shot echo planar T2*-weighted image sequence with the following parameters: TR = 2000 ms; volumes = 150; TE = 30 ms; FA = 90°; matrix size = 64 × 64; voxel size = 4 × 4 × 4 mm^3^; 32 slices, flip angle = 9°, FOV = 256 × 256 × 252 mm, phase encode A-P, Bandwidth = 2442/px. Participants were asked to keep their eyes closed and to refrain from moving to limit the induction of motion artifacts as much as possible to the scans.

### MRI analysis

Functional images were pre-processed using fMRIPrep version 20.2.7_LTS [57], which is based on Nipype version 1.7.0 pipeline [58]. This pipeline combines software from several neuroimaging packages (Nilearn version 0.6.2, ANTS version 2.3.3 [59], FSL version 5.0.9 [60], FreeSurfer version 6.0.1, and AFNI version 16.2.07 [61]. The following description of fMRIPrep’s pre-processing is based on boilerplate provided with the software covered by a no rights reserved (CC0) license. For more details of the pipeline, visit the section corresponding to workflows in fMRIPrep’s documentation.

Structural T1-weighted (T1w) images were corrected for intensity non-uniformity with N4BiasFieldCorrection [62], distributed with ANTs 2.3.3, and used as T1w-reference throughout the workflow. The T1w-reference was then skull-stripped with a Nipype implementation of the antsBrainExtraction.sh workflow (from ANTs), using OASIS30ANTs as target template. Brain tissue segmentation of cerebrospinal fluid (CSF), white-matter (WM) and gray-matter (GM) was performed on the brain-extracted T1w using FSL fast. Brain surfaces were reconstructed using recon-all, FreeSurfer 6.0.1, and the brain mask estimated previously was refined with a custom variation of the method to reconcile ANTs-derived and FreeSurfer-derived segmentations of the cortical gray-matter of Mindboggle [63]. Spatial normalization to the standard space (MNI152NLin2009cAsym) [64] was performed through nonlinear registration with antsRegistration (ANTs 2.3.3), using brain-extracted versions of both T1w reference and the T1w template.

For each of the two BOLD runs of each participant, the following preprocessing was implemented. A skull-stripped reference BOLD volume was generated through fMRIPrep. A B0-fieldmap was estimated based on a phase-difference map calculated with a dual-echo GRE (gradient-recall echo) sequence, processed with a custom workflow of SDCFlows inspired by the epidewarp.fsl script and further improvements in HCP Pipelines [65]. The fieldmap was then co-registered to the target echo-planar imaging (EPI) reference run and converted to a displacements field map with FSL’s fugue and SDCflows tools. Based on the estimated susceptibility distortion, a corrected EPI reference was calculated for a more accurate co-registration with the anatomical reference. The BOLD reference was then co-registered to the T1w reference using bbregister (FreeSurfer) which implements boundary-based registration [66]. Head-motion parameters were estimated before any spatiotemporal filtering using mcflirt (FSL 5.0.9). BOLD runs were further slice-time corrected using 3dTshift from AFNI [61]. The BOLD time-series were then resampled onto their original, native space by applying a single, composite transform to correct for head-motion and susceptibility distortions. The BOLD time-series were resampled into standard space, generating a preprocessed BOLD run in MNI152NLin2009cAsym space. Gridded (volumetric) resamplings were performed using antsApplyTransforms (ANTs), configured with Lanczos interpolation to minimize the smoothing effects of other kernels [67]. Non-gridded (surface) resamplings were performed using mri_vol2surf (FreeSurfer).

Quality control of functional images was assessed using the fMRIPrep’s visual reports. Data were visually inspected for whole-brain field of view coverage, and proper alignment of the functional image to the corresponding anatomical reference. All images passed the quality control procedure.

### Functional networks preprocessing

We used load_confound and NiftiLabelsMasker functions from Nilearn (version 0.10.1)[68] in Python (version 3.10.2) to extract the timeseries from each cortical region of the Schaefer7–400 atlas parcellation [43]. High pass filtering was applied using Load_confound, which adds discrete cosines transformation basis regressors to handle low-frequency signal drifts. The average signal of the white matter (WM) and cerebrospinal fluid masks (CSF), and motion parameters with their first-degree derivatives (translation, rotation parameters plus their derivatives with a sum of 12 parameters), were included in Load_confound to be regressed out from the BOLD signal. A scrubbing approach was applied to remove frames with excessive motion as determined by the FD > 0.5, and temporal Derivative of root mean square VARiance over voxels (DVARS) > 3. For scrubbing, if fewer than five contiguous volumes had unscrubbed data, these volumes were also scrubbed and interpolated [69]. Resting-state fMRI runs with less than 70 volumes were excluded and participants were excluded from our analysis if both runs were excluded with respect to this criterion. Accordingly, among participants scanned for both PET and rs-fMRI, 218 individuals satisfied these conditions and were further included in our analysis. Masker.fit_transform and NiftiLabelMasker in Nilearn were used to apply low pass filtering with a cut-off set at 0.08 HZ, to smooth the images using a 6mm Guassian kernel full width half maximum, to extract the timeseries for each of Schaefer7–400 atlas regions [43], and to z-score the extracted timeseries. For participants with both runs available, we concatenated the extracted BOLD signal timeseries. The average FD values was computed for each fMRI scan run. If the two runs were accepted for a scan, the FD was averaged across both runs. These averaged FD values were incorporated as covariates in our statistical models.

### Static functional connectivity computation

SFC captures how an extracted BOLD timeseries of a brain region is activated in correspondence to the timeseries of other regions. To estimate sFC, we calculated the Pearson correlation between the z-scored timeseries of all pairs of Schaefer atlas regions, resulting in a 400⋅400 functional connectivity matrix. Functional connectivity matrices were thresholded to retain only positive values [70]. Average of functional connectivity estimates were computed for within and between each of the seven Schaefer7–400 atlas and between networks.

### Time-varying functional connectivity computation

TVFC was computed using the edge timeseries method [28]. Considering $x_{i}=\left[x_{i}(1),\ldots ,x_{i}(T)\right]$, and $x_{j}=\left[x_{j}(1),\ldots ,x_{j}(T)\right]$ as two z-scored timeseries extracted from brain parcels i and j (T representing the length of the timeseries), the Pearson correlation coefficient of the two timeseries was computed as $r_{ij}=\frac{1}{T-1}\sum_{t=1}^{T}\left[x_{i}(t).x_{j}(t)\right]$. N corresponds to the number of brain functional parcels and repeating this procedure for all the pairs of parcels results in a [N×N] dimension functional connectivity matrix. By skipping the averaging part of the mentioned procedure (not exactly an average, as we divided by T-1 rather than T), results in an element wise product of the two timeseries i and j as the co-fluctuation of the two timeseries in time. This procedure generates a vector of length T whose elements represent the moment-by-moment co-fluctuation magnitude of parcels i and j. Repeating the same operation for all pairs of the parcels, provides a [N×N] dimension co-fluctuation matrix per each timepoint of the scan session (TR) resulting in a tensor of size [T×N×N]. If the activities of parcels i and j are simultaneously positive or negative, the co-fluctuation is positive but if one is positive and the other is negative or vice versa, it would result in a negative co-fluctuation value. Also, if an activity of a parcel is close to zero, the product of the two will also be close to zero [28]. The obtained co-fluctuation matrices reflect similarity/dissimilarity in the activation of pairs of brain parcels in time. For each participant, the upper triangle of the co-fluctuation matrices was vectorized at all time points. Next, the similarity of the two consecutive co-fluctuation vectors was obtained using the Pearson correlation and stored in a vector size T-1 (Fig. 1b). To identify the variability in co-fluctuations across time, we computed the coefficient of variation of the similarity vectors. All the analysis in this section was performed using Matlab version R2021b. A brief representation of the process is illustrated in Fig. 1b.

We compared TVFC estimated from the empirical data to that derived from a time-shuffled surrogate model. The time-shuffled surrogates were generated by randomly permuting the order of co-fluctuations in the TVFC stream while keeping individual co-fluctuations unchanged. This approach preserved the mean and variance of each co-fluctuation matrix but disrupted sequential correlations, thereby reflected the null hypothesis that no temporal structure existed in the TVFC stream. For each participant, 1,000 surrogate realizations were generated and the TVFC metric was computed for each realization, yielding a subject-specific null distribution. Empirical TVFC values were then evaluated against their corresponding null distributions using a two-sided Monte Carlo test with finite-sample correction, providing subject-level p-values that quantify the deviation of empirical TVFC values from their corresponding null distributions.

### Neuropsychological assessments

Participants underwent yearly cognitive assessments using one of the four versions of the RBANS [46]. The RBANS provides scaled scores across five cognitive domains, including immediate and delayed memory, attention, visuospatial construction skills, and language, offering a comprehensive assessment of neurocognitive function. We further performed our analysis on the RBANS total composite score. Cross-sectionally, we used the cognitive assessment closest in time to each participant’s fMRI scan to evaluate associations between functional connectivity measures and cognition. Longitudinally, cognitive changes were evaluated by calculating linear slopes from RBANS scaled scores using linear mixed effect models, incorporating all available cognitive assessments conducted before or after the fMRI visits (mean number of longitudinal cognitive assessments per participant = 7.3, SD = 2.3).

### Statistical analysis

We performed linear regression models to assess the relationship between baseline and longitudinal AD proteinopathy and cognitive measurements with TVFC and sFC. Age, sex, the time interval between the PET and fMRI scan. as well as the average of the FD, were added as covariates in all models assessing associations between the functional connectivity measures and pathology. Linear mixed effect models were used to extract the slopes of cognition change across multiple time points of cognitive data acquired. For associations between the functional connectivity measures and cognition (cross-sectionally and longitudinally) sex, years of education and average FD were considered as covariates in our linear regression models (age was already taken into account with respect to the cognitive indices used). To assess the potential effect of Aβ on the relationship of sFC and TVFC with tau and cognition, Aβ was added as an interaction term with our connectivity measures to our linear regression models. Model performances were compared using the AIC, which balances model fit and complexity. The network with the lowest AIC value and highest beta estimate was interpreted as the one representing the strongest association. When expanding our analysis to all networks, FDR correction was applied to account for the twenty-eight within and between resting-state networks statistical tests, and our findings were further reported with and without the applied correction.

### Time shuffled TVFC null model

We compared TVFC estimated from the empirical data to the one derived from a time shuffled surrogate model to investigate the robustness of the reported analysis. The time-shuffled surrogates were generated by randomly permuting the order of co-fluctuations in the TVFC stream while keeping individual co-fluctuations unchanged (Fig. 4c). This approach preserves the mean and variance of each co-fluctuation matrix but disrupts any sequential correlations. As a result, time-shuffled surrogates align with the null hypothesis that no sequential correlations exist in the TVFC stream. For each participant, we generated 1,000 surrogate realizations and computed the TVFC metric for each realization, yielding a subject-specific null distribution. Empirical TVFC values were then compared to their corresponding surrogate distributions using a two-sided Monte Carlo test, where p-values were calculated as the proportion of surrogate values exhibiting equal or greater deviation from the surrogate mean than the empirical estimate (with finite-sample correction). This procedure provided subject-level p-values quantifying the extremeness of empirical TVFC relative to its null distribution which we reported the median of these subject-level p-values.

To determine whether the observed association between TVFC and longitudinal tau accumulation depended on the genuine temporal structure of the data, the empirical standardized regression coefficient for each network pair was compared against a null distribution of coefficients obtained from surrogate TVFC model. Specifically, the identical group-level regression model was refit 1,000 times using time-shuffled TVFC estimates, generating a surrogate-derived distribution of standardized beta coefficients. The empirical beta was then evaluated against this null distribution using a two-sided Monte Carlo test, with p-values calculated as the proportion of surrogate betas with absolute magnitude greater than or equal to the empirical estimate (with finite-sample correction). This analysis quantified whether the strength of the association exceeded that expected under temporally shuffled TVFC. The same surrogate-based regression procedure was applied to the TVFC × baseline Aβ interaction models predicting longitudinal cognition. For each network pair, the standardized interaction coefficient from the empirical model was evaluated against a null distribution of interaction coefficients obtained by refitting the identical model using 1,000 time-shuffled surrogate TVFC estimates, and Monte Carlo p-values were computed as described above.

## Supplementary Material

Supplementary Files

This is a list of supplementary files associated with this preprint. Click to download.

• SupplementaryMohammadaliJavanray.docx

## Figures and Tables

**Table 1:** Demographics

PREVENT-AD sample demographics and clinical characteristics	
Sample size	218
Age in years	68.16(5.19)
Female, %(n)	72.48(158)
Years of education	15.51(3.27)
APOE ε4 carriers, % (n)	39.91(87)
Global Aβ SUVR (baseline)	1.32(0.29)
Temporal meta-ROI tau SUVR (baseline)	1.16(0.12)
RBANS total scale index (baseline)	104.07(10.07)
Years between fMRI and baseline PET scans	2.87(2.20)
Years between baseline and follow up Aβ PET scans	4.33 (0.54)
Years between baseline and follow up tau PET scans	4.28(0.55)
Number of cognitive visits	7.33 (2.30)
Time interval between cognitive visits	1.23(0.75)
Duration of cognitive follow up in years	7.81(1.82)

## Data Availability

Data used in this study were obtained from the Presymptomatic Evaluation of Experimental or Novel Treatments for Alzheimer’s Disease (PREVENT-AD, https://prevent-alzheimer.net/). Portions of the dataset are openly accessible through the PREVENT-AD Open Science repository (https://openpreventad.loris.ca/). In accordance with the PREVENT-AD ethical and privacy framework, which ensures participant confidentiality and their right to restrict open sharing of certain biological or imaging data, the complete internal release of the dataset can only be shared upon approval by the scientific committee at the Centre for Studies on Prevention of Alzheimer’s Disease (StoP-AD) at the Douglas research centre. Information about the data access process for both the open and full internal releases is provided at https://prevent-alzheimer.net/?page_id=1760&lang=en. Further details about the PREVENT-AD dataset’s structure and content can be navigated through Villeneuve et al., 2025 [27] and Tremblay-Mercier et al., 2021 [45]. For questions regarding data access, please contact co-author S. Villeneuve (sylvia.villeneuve@mcgill.ca).

## Code availability

fMRI data were preprocessed using fMRIPrep (LTS v20.2.7) [57]. Post-processing denoising was performed in Python (version 3.10.2) using Nilearn (version 0.10.1)[68], where confound regression was applied via the load_confounds module and regional timeseries were extracted with NiftiLabelsMasker from the Schaefer 7–400 and 7–200 parcellations. All timeseries were subsequently z-scored. Computation of TVFC, sFC, and all surrogate TVFC models was carried out in MATLAB (R2021b). Statistical analyses were performed in R (version 4.3.1), except for the Monte Carlo tests, which were implemented in MATLAB. Longitudinal cognitive slopes were estimated using linear mixed-effects models implemented in the lme4 package (version 1.1–37), and linear regressions and group-comparison visualizations were generated using ggplot2 (version 3.5.2). Null-model visualizations and timeseries/functional connectivity matrix plots were produced in MATLAB. The scripts used to implement the statistical analyses presented in this manuscript are available in an open-access GitHub repository: https://github.com/javanray/Longitudinal_pathology-FC.git
